# Riksmaten Adolescents 2016–17: A national dietary survey in Sweden – design, methods, and participation

**DOI:** 10.29219/fnr.v62.1381

**Published:** 2018-08-28

**Authors:** Lotta Moraeus, Eva Warensjö Lemming, Ulla-Kaisa Koivisto Hursti, Marianne Arnemo, Jessica Petrelius Sipinen, Anna-Karin Lindroos

**Affiliations:** 1 Department of Risk and Benefit Assessment, National Food Agency, Uppsala, Sweden; 2 Department of Surgical Sciences, Orthopaedics, Uppsala University, Uppsala, Sweden; 3 Department of Internal Medicine and Clinical Nutrition, Institute of Medicine, Sahlgrenska Academy, University of Gothenburg, Gothenburg, Sweden

**Keywords:** Children, adolescents, dietary assessment, web-based, biomarkers, national sample

## Abstract

**Background:**

Nationally representative information on food consumption data is essential to evaluate dietary habits, inform policy-making and nutritional guidelines, as well as forming a basis for risk assessment and identification of risk groups.

**Objective:**

To describe the methods used in the Swedish national dietary survey of adolescents, Riksmaten Adolescents 2016–2017.

**Design:**

Students in grades 5, 8, and 11 (mean ages 12, 15, and 18 years) were recruited in this school-based cross-sectional survey. A new, validated, web-based method was used to assess dietary intake. Information on physical activity, health, and socioeconomic background was collected through web questionnaires. Physical activity was also evaluated by accelerometers. Weight and height were measured in all participants, while blood and urine samples were collected in a subsample of 40% of the participants.

**Results:**

A total of 3,477 (68%) respondents participated and 3,099 (60%) had complete dietary information. In the subsample, 1,305 (55%) respondents participated and 1,105 (46%) had complete dietary information. The participants were overall representative for the population with regard to socioeconomic background and school organization (public or independent). All types of municipalities were represented in the survey and overall, the geographic distribution corresponded to the underlying population. Some differences by school grade were observed. Sample weights were calculated for the total sample and the subsample.

**Conclusion:**

The Riksmaten Adolescents 2016–2017 provides valuable national data on diet, physical activity, and markers of exposure in age groups where data have been lacking. The data will provide a valuable basis for risk assessment, public health policy, and in-depth analyses.

## Introduction

National dietary surveys provide essential information for developing public health policy and food regulations. They also provide data for future comparisons of food intake and dietary habits. The latest national surveys performed by the National Food Agency (NFA) in Sweden were Riksmaten Children 2003 (age groups 4, 8, and 11 years), and Riksmaten Adults 2010–2011 (1, 2). Corresponding data on adolescents in Sweden are so far missing.

Dietary surveys provide information on nutrient and energy intake, food choices, and intake of unwanted substances, such as contaminants and natural toxins, which may be present in food. It is especially important to track children’s food consumption since their growth and well-being require foods of high nutritional quality. Furthermore, there is evidence that children are more sensitive to certain toxic compounds than adults (3). In the Riksmaten Children 2003 survey, 25% of children’s energy intake was derived from foods high in energy and low in nutrients, such as soft drinks, sweets, ice cream, and crisps. Further, children consumed only half of the recommended amount of fruit and vegetables (1). Children who reported eating school lunch every day had significantly higher intake of vitamin D and folate, even after adjusting for energy intake (4). However, analyses of reported school meals uncovered that they did not reach the recommended levels of dietary fiber or vitamin D and E (5). Many efforts have since then been made to improve the diet in children, often with focus on school meals. Since 2011, the *Swedish Education Act* (SFS 2010:800) dictates that in addition to being free of charge, the meals should be nutritionally balanced. The NFA has supported implementation of the law by providing guidelines to school meal planners based on the Nordic Nutrition Recommendations (6). These include offering a salad bar with at least five varieties of vegetables and banning snacks, soda, and sweets from school canteens (7).

Dietary and biomonitoring data from national surveys are also important as a basis for the scientific input to risk managers at the NFA. In Riksmaten Adults 2010–2011, blood and urine were collected in a subsample which have been used for risk–benefit assessments, such as risk–benefit assessment of consumption of nuts (8) and fish (9, 10). The data can also be used in modeling of exposure scenarios as a scientific base for weighing of effects of different risk management options on human health risks (11). Biomonitoring data are important in assessment of nutritional status for certain nutrients such as vitamin D, folate, iron, and iodine (12, 13). In addition, data are used for assessment of total human exposure to contaminants, for example, heavy metals, polychlorinated biphenyls (PCBs) and perfluorinated alkyl acids (PFAA) in blood and urine (14–16).

Results from dietary surveys also form the basis for decisions on food fortification and common food legislation on a European level. Without country-specific data, it is difficult to influence these guidelines and regulations.

One of NFA’s overall goals is to improve the diet in those who have unhealthy diets. In order to meet this goal, there is a need to identify risk groups in the population. With Riksmaten Adolescents 2016–2017, there will be a possibility to investigate diet in relation to a number of background factors, such as sex, age, and sociodemographic background, in a large representative sample.

## Aim

The aim of this paper is to describe the methods of the national dietary survey Riksmaten Adolescents 2016–2017 and its add-on survey Riksmaten Adolescents Plus. Furthermore, it aims to present a drop-out analysis at school and student level.

## Design, subjects, and methods

### Study design

Riksmaten Adolescents 2016–2017 is a nationally representative cross-sectional school-based dietary survey of children and adolescents. It was carried out in Sweden in school grades 5 (mean age 11.5 years; SD 0.42), 8 (mean age 14.5 years; SD 0.42), and 11 (mean age 17.7 years; SD 0.66) between September 2016 and May 2017. The survey included a web-based dietary assessment method, web questionnaires, measured weight and height and objectively measured as well as self-reported physical activity. In addition, blood and urine samples were collected in a subsample of participants. Riksmaten Adolescents Plus was an add-on to the main survey with the aim to include adolescents who did not attend school but were in the age group of school grade 11. Riksmaten Adolescents Plus will be described in a separate section at the end of the methods section. Ethical approval for the main and add-on surveys was obtained from the Regional Ethical Review Board in Uppsala (No. 2015/190).

### Population and sampling

A total of 619 schools, approximately 200 from each school grade, were selected by Statistics Sweden (SCB) to represent students in the three age groups. Schools were sampled on type of municipality which is a classification by the Swedish Association of Local Authorities and Regions, based on structural factors such as population and commuting patterns (17). Sampling was also based on whether the school was public or charter school (independent schools with public funding) as well as geographical spread. Schools with fewer than 10 students in a school grade and high schools with only language introduction classes were excluded. Forty percent of schools were randomly selected for blood and urine sampling. The biological sampling was conducted by regional divisions for Occupational and Environmental Medicine (AMM) and thus each school in the sample was allocated to one AMM division based on geographical location. To get an even distribution over the year from different parts of Sweden all the regions were visited both in the spring and in the autumn. For logistic reasons, the schools without biological sampling were also allocated to AMM regions and recruited simultaneously.

**Fig 1:**
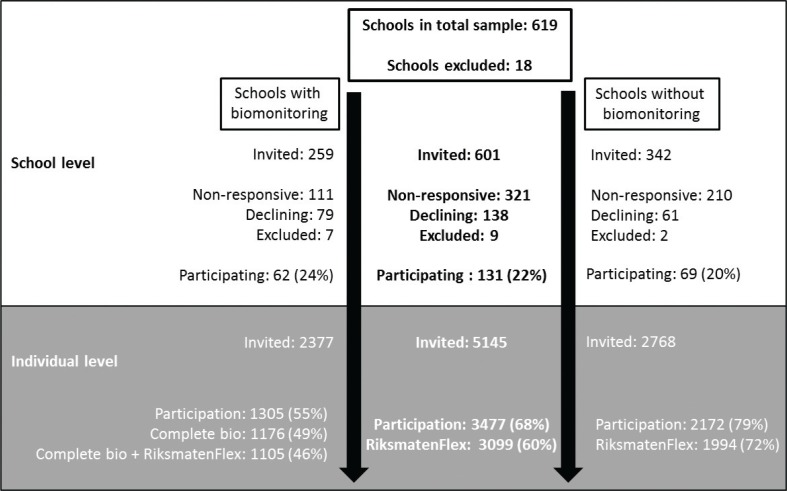
Inclusion process and participation at school and student level. Bio: biological sampling.

Contact information of principals was collected through school or municipality websites or by phoning the school reception. At this stage, 18 schools were excluded from the sample due to too few students; they only provided language introduction classes or the school had closed (Fig. 1). Schools were then contacted through an invitation email addressed to the principal. Principals who did not reply to the email were contacted by telephone. Since the goal was to include equal number of participants from each school grade, recruitment was rotated between the school grades. One or two classes were included from each school and when the desired number of classes was recruited in a school grade, no further schools in that school grade were contacted. Names and addresses of students in the included classes were collected from the schools and information letters were sent out to students’ legal guardians 3 weeks before the school visit. For schools with blood and urine sampling, consent forms for all students, and guardians of children younger than 16 years, were included in the letters. The written consent was collected by teachers and provided to the research team at the day of the school visits. In schools without biological sampling, the ethics committee approved the request to use opt out consent.

Prior to the data collection, the class teacher was asked to show a short information film to the students. This was a complement to the written information and the information given at the school visit.

## Data collection

The school visits took place during school hours and normally started in the morning. Each class was visited a certain day with a few exceptions where blood and urine samples were collected the day before or after. The school visits were similar for grade 5, 8, and 11, but additional time was added in grade 5 visits. Field staff from the NFA was responsible for planning the visits in cooperation with school staff. During the school visits, NFA staff informed students about the survey and instructed them on the web-based method. They also weighed and measured the participants, except in schools with biological sampling, where AMM was responsible for measurements and collecting blood and urine samples. After blood and urine sampling, the participants were offered a snack of juice and fruit and were able to rest if needed.

During the school visit, participants registered their dietary intake and completed questionnaires on dietary habits and lifestyle through the web-based RiksmatenFlex, described below. The web-based nature of RiksmatenFlex required that all participants had access to a computer, tablet, or smart phone with an Internet connection, both at school and in their homes. In 2016, a majority of Swedish households (93%) had Internet access and in the age group of 16–54 years, 97% had access (18). In the few cases where schools could not provide a computer or tablet, participants could borrow a tablet from NFA or complete the registration on a smart phone. If the questionnaires and/or dietary registration were not completed, automatic reminders were sent to the email and phone number provided by the participant when they first logged on to the website. Participants received a gift certificate valued at 200 SEK or 300 SEK (the subsample with biological sampling) after full participation.

Prior to the survey, NFA field staff trained together in order to standardize the data collection. Information about the survey and instructions on RiksmatenFlex were given in a similar manner during the school visits and any questions from participants were answered according to a question and answer guide. After each school visit, a rough data check was performed in order to confirm that no errors had occurred during the web registration.

### RiksmatenFlex

A new web-based method, RiksmatenFlex, was developed by the NFA for Riksmaten Adolescents 2016–2017. RiksmatenFlex is a website that includes a dietary registration part based on the repeated 24-h multiple pass recall method (RiksmatenFlexDiet) and questionnaires (RiksmatenFlexQ). The content is flexible and can be adjusted to fit different surveys. The web site is compatible with most modern web browsers and could be accessed through computer, tablet, or smartphone. The method was validated before the main survey by comparing two 24-h recalls in RiksmatenFlexDiet with two 24-h recall interviews and biomarkers. The results show that RiksmatenFlexDiet provides information on intake of energy, fruit, vegetables, and wholegrains that is at least as valid as that from 24-h recalls (AK Lindroos, personal communication, May 29, 2018).

#### RiksmatenFlexDiet

The European Food Safety Authority (EFSA) recommends the use of 24-h recall that includes two non-consecutive days (19). Participants in the current survey registered 3 days of intake whereof recall day one and three were non-consecutive and retrospective. The first recall day was always the day before the school visit and was registered in the class room. The second day was the day of the school visit and was started in the class room and finished the next day at the latest. The third day was restrospectively registered and randomly assigned to occur 2–7 days after the first recall day and was a weekday (Monday through Thursday) or weekend day (Friday through Sunday), depending on the first recall day. The participants were able to see the date of the third day but the registration form was closed until participants were prompted via a text message and email to register their intake the day before. The aim was to get a proportional representation of all weekdays. Participants with complete registration of at least the first and third recall days in RiksmatenFlexDiet will be included in the dietary sample used in the main results report. The second recall day is excluded from analyses due to the mixed method and the fact that offering fruit and juice to participants with biological sampling could influence the food intake. However, it is possible to include the second recall day in other analyses when appropriate.

Based on experiences from the previous web-based method used in Riksmaten Adults 2010–2011, the number of foods was reduced and more generic foods were included. The generic foods were created as a complement to the specific foods, for example, meatballs which were based on a combination of different kinds of meat. There was a possibility to refine the items in a second step. Thus, 778 food items were available to choose from in the first step of the registration but adding information about type of meat, berry, fish, sweetener, and so on in the second step, resulted in more than 2,300 items. The food items were connected to the Swedish national food composition database (version Riksmaten Adolescent 2016–2017) and energy and nutrient intakes were calculated automatically in the database.

During the registration, participants chose the time of their meals from a calendar view. They were then asked to specify type and location of each meal. Food items were added by using the search field and choosing from the food list. It was possible to add single ingredients or complete meals. In a second step, participants indicated amount of food and specified the food item, if possible. A picture portion guide, household measures, pieces and slices, and so on were used to estimate the amounts eaten. The picture portion guide was embedded in the RiksmatenFlexDiet interface and included 39 different food categories, with 4–8 different reference sizes in each category. Six reference sizes were most common. Portion pictures of glasses and cups were also included. In addition, RiksmatenFlexDiet included five different picture series (breakfast cereals, breads, sandwiches, fat spreads, and ice creams) containing 4–16 different typical foods in each food category to facilitate choosing the correct food eaten.

Throughout the registration, participants were reminded automatically of foods easily forgotten and were prompted to review their registered foods before signing off the registration as complete. Once the registration of a day was signed off, it could only be opened by NFA staff. At the school visit, field staff checked the first recall day for completeness before students were asked to sign it off.

#### RiksmatenFlexQ

Three questionnaire items were filled out during the school visit: ‘You and the family’, ‘Eating habits’, and ‘More eating habits’. There was also a ‘Parental questionnaire’ which contained questions that could be difficult for the participants to answer, such as duration of breast feeding and parental education. Participants were therefore instructed to ask their parents to fill out the questionnaire at home. Participants were however able to complete the ‘Parental questionnaire’ by themselves, which was done by 6% in school grade 5, 18% in school grade 8, and 40% in school grade 11. In these cases, careful consideration has to be taken when analyzing specific questions.

#### Food consumption

The questionnaires ‘Eating habits’ and ‘More eating habits’ contained questions about dietary habits and food consumption. In ‘Eating habits’, participants answered questions about specific food preferences, type of fat used on bread, and whether organic milk was consumed in the household. Both questionnaires contained non-quantitative food propensity questionnaires (FPQ) to capture food items that were consumed less frequently. Foods such as vegetables, fruits, berries, meat, and fish were included as well as nuts and mushroom together with consumption of snacks, fast food, and beverages.

Questions about eating habits were also addressed in the questionnaire titled ‘You and the family’. Participants were asked whether they regularly consumed dietary supplements and if so, what they were and how often they were consumed. They were also asked on how many school days they ate school meals and if and how they substituted any skipped meals. The ‘Parental questionnaire’ contained questions about type of salt, type of cooking fat, and percentage of organic vegetables consumed in the household.

#### Physical activity

In ‘You and the family’, students were asked to state whether they participated actively during physical education in school and their method of transportation to school. Further, they described their general activity level during the last week on a four-graded scale from sedentary activities to intense exercise and competition. Finally, they were asked whether they participated in organized activities and if so which activities and how often during a week. Physical inactivity was addressed in four questions including reading and screen time.

#### Individual characteristics of child and parents

In the questionnaire ‘You and the family’, participants answered questions about their state of health and use of medicines, family structure, country of birth (child and parents), as well as about how they perceived their health and weight. Further, girls were asked whether, and if so at which age, they had had their first period. Participants in grade 8 and 11 were asked about their alcohol and tobacco use. Information on parental occupation and education was included in the parental questionnaire.

### Measurement of physical activity

Accelerometers Actigraph GT3X and GT3X+ were used to objectively measure physical activity among participants. The accelerometer was distributed during the school visit and participants were instructed to use them for 7 days except at night and when exposed to water. ActiLife version 6.12.0 was used to download the data at a sample frequency of 30 Hertz and epoch of 5 sec. The accelerometer measures duration and intensity of physical activity and gives an output of activity counts per epoch. Counts per minute (cpm) can be calculated and used to estimate moderate to vigorous physical activity (MVPA) according to international cut-points.

### Anthropometrics

Height and weight were measured by the trained staff from NFA or AMM during school visits. Standardized methods (20) and field equipment were used. Participants generally wore light clothing and no shoes and when heavier clothing was worn, this was recorded. Weight was measured to the nearest 0.1 kg using SECA 862 or 899 digital weighing scales. Height was measured to the nearest 0.1 cm using SECA 213 portable stadiometers.

### Blood and urine samples

The biological sampling was conducted by regional divisions for Occupational and Environmental Medicine (AMM). The AMM teams consisted of 3–4 members whereof one was responsible for planning the school visits in collaboration with the NFA. The samplings were conducted non-fasting in the school health facilities or equivalent at the school premises. The blood samples were centrifuged immediately and pipetted into aliquots and placed in freezers together with the urine sample. All samples were transported to the AMM division where they were stored in −80°C. When a region had completed the sampling in all schools, the samples were transported to NFA in Uppsala, Sweden, and stored in −80°C.

### Riksmaten Adolescents Plus

Around 10% of the age group in school grade 11 in Sweden does not attend school, and an additional aim was to reach and collect data on these adolescents. The ‘Municipal activity responsibility’ (KAA) is required to keep records of these adolescents but there are substantial differences in procedures in different municipalities, and lists of adolescents are updated continuously. Thus, a convenience sample approach was adopted and study participants were recruited through KAA. The KAA are responsible for adolescents aged 16–19 years, and for logistic reasons the target group was extended to that age group. The person responsible for KAA in municipalities with at least 10 adolescents in the target group was contacted via email and telephone. The goal was to include 100 participants and even though the aim was not to collect a representative sample, participants were recruited from rural as well as urban municipalities throughout Sweden.

The data collection was similar to the main survey but differed in some key aspects. The invitation letter was not posted to the participants but provided to them by the KAA staff. The data collection took place in facilities provided by KAA and included one participant at a time or smaller groups. The questionnaires were slightly modified to suit the target group by removing school-related questions. There was no blood and urine sampling but anthropometric measurements and accelerometer data were collected. Data from Riksmaten Adolescent Plus will be compared to adolescents in grade 11 and can be combined with the total sample when appropriate.

### Data quality

Data on weight, height, and biological samples were double-checked by field staff after each school visit. Further, recall days with less than 800 kcal or more than 3,500 kcal were checked manually according to pre-specified criteria in order to detect erroneous energy intake. Days with more than two meals and five foods were approved, as well as days marked as unusual by the participant. High intakes were checked for feasibility. Unclear registrations were discussed in the research group before deciding whether to reject the registration; 21 recall days were rejected at this stage. All recall days that included an energy intake of more than 800 kcal but had not been indicated as finished were also checked to avoid omitting registrations that were complete but not signed off as finished in the system. In addition to the above criteria, at least one meal or food had to be registered after 2 pm. One hundred and three recall days that had not been signed off by the participants were considered to be complete. The data quality check was performed similarly in the main survey and Riksmaten Adolescent Plus. Only two recall days were rejected and five that had not been indicated as finished were approved in the add-on survey.

### Drop-out analysis

SCB was responsible for performing a drop-out analysis where participating schools and students were compared to the population on relevant variables. The analysis was delivered in a technical report in Swedish to the NFA. The population was the total of schools in Sweden with school grades 5, 8, or 11. School organization and type of municipality were used as sampling variables and were expected to be representative of the population. Municipalities were categorized into one of five groups by SCB based on the classifications by Swedish Association of Local Authorities and Regions (21): 1) metropolitan municipalities, 2) suburbs surrounding metropolitan areas, 3) larger cities and surrounding suburbs, 4) densely populated municipalities, and 5) other municipalities (commuter municipalities, tourism and travel industry municipalities, manufacturing municipalities, sparsely populated municipalities, and municipalities in sparsely populated regions). School size was also a sampling variable but since smaller schools were undersampled by design, these were not expected to be representative on school level. Further, participants’ personal identity numbers were collected during the survey and were used by SCB to connect survey data to background variables from different registers. The highest disposable income of either the mother or father was collected from the Income and Tax Register and categorized into 1) <450,000 SEK or unknown, 2) 450,000–649,999 SEK and 3) ≥650,000. The highest attained education level of either the mother or the father was collected from the Education Register and categorized into 1) primary school or unknown, 2) high school or 3) university degree. Country of Birth was based on both the student and their parents and was categorized into Sweden (student and at least one parent born in Sweden) or other country (student born abroad or both parents born abroad). Sex and school grade (5, 8, or 11) of participants were also used in the drop-out analysis.

To assure that data from Riksmaten Adolescent 2016–2017 would be representative, SCB also computed two sets of sample weights, one for all participants and one for the subsample. The sample weights should correct for any deviations between the participants and the population, and were based on school grade, type of municipality, school organization, school size and sex. School organization was excluded when calculating sample weight for the subsample.

## Results

Overall, 131 schools throughout the country agreed to participate in the survey, 72 during the fall 2016 and 59 during spring 2017 (Fig. 1). Schools that were non-responsive include 146 schools that were only contacted by the invitation email and 175 schools that could not be reached by telephone after the initial email invitation. In total, 3,477 individuals (68%) participated in some stage of the survey. A total of 3,099 participants (60%) had complete diet information from at least the first and third recall day with higher response rate in grade 5 (67%) than in grade 8 (58%) and grade 11 (57%). In the subsample, the response rate was 55% overall and 46% had complete biological samples as well as diet information (Fig. 1). The response rates were similar across school grades: 48, 47, and 45% with complete information in grades 5, 8, and 11, respectively. A majority completed all questionnaires, and anthropometric measurements were available for almost all participants (Table 1). Males and females participated to an equal degree in school grade 5 but in school grade 11, 56% of participants were female (Table 2). All types of municipalities were represented (data not shown) and the distribution was similar in all school grades with some exceptions (Table 2). A larger proportion of students in grade 11 attended schools in ‘other municipalities’ compared to school grades 5 and 8. In school grade 5, 25% attended schools in ‘suburbs surrounding metropolitan areas’ compared to around 10% of the older participants.

**Table 1:** Participation on student level in different stages of survey

	*n*	
**Potential participants according to class list**	5,224	
Had moved	42	
Excluded^a^	36	
**Total sample**	*n*	% of eligible
Eligible participants	5,145	
Declined	1,669	32
Participated in some stage of survey	3,477	68
RiksmatenFlexDiet	3,099	60
	*n*	% of participants
Information on age	3,475	99.9
Height and weight measurements	3,450	99
Completed all questionnaires	3,157	91
Education level from parental questionnaire	3,003	86
**Biological sample**	*n*	% of eligible
Eligible participants	2,378	
Declined	1,073	45
Participated in some stage of survey	1,305	55
Complete biological sampling	1,176	49
RiksmatenFlexDiet + biological sampling + height and weight measurements	1,105	46

a Participated in language introduction/special education/adult education.

**Table 2:** Age, sex, and geographical distribution of participants according to school grade

	Total *N* = 3,477	School grade 5 *N* = 1,217	School grade 8 *N* = 1,198	School grade 11 *N* = 1,062
Age, years (min; max) (*n* = 3,474)	15 (10; 21)	12 (10; 15)	15 (12; 17)	18 (16; 21)
**Sex**				
Female	1,842 (53)	622 (51)	625 (52)	595 (56)
Male	1,635 (47)	595 (49)	573 (48)	467 (44)
**Type of municipality *n* (%)**				
Metropolitan	471 (14)	101 (8)	220 (18)	150 (14)
Suburbs surrounding metropolitan areas	483 (14)	303 (25)	84 (7)	96 (9)
Larger cities and surrounding suburbs	1,425 (41)	458 (38)	593 (50)	374 (35)
Densely populated	301 (9)	90 (7)	153 (13)	58 (5)
Other municipalities	797 (23)	265 (22)	148 (12)	384 (36)

The expected proportion of recall days in week days and weekend days was achieved, with 56% of recall days Monday through Thursday and 44% on Friday through Sunday. Mean registered meals per day was lowest on Sundays (4.2 meals) and highest on weekdays (4.8 meals).

In the add-on survey Riksmaten Adolescents Plus, 100 participants were included and 81 had registered at least recall day one and three. The results from the add-on survey will not be further addressed in this article.

### Participation in relation to the population

Due to the undersampling of smaller schools, the distribution on school level was skewed toward larger schools as compared to the population (Table 3). This was mirrored in the under-sampling of schools with school grade 5 and oversampling of school grade 11. Younger students were however more likely to participate, resulting in 1,217 students in school grade 5, 1,198 in grade 8, and 1,062 in school grade 11, respectively; a distribution comparable to the population (Table 4).

**Table 3:** School-level characteristics in total sample, subsample, participating schools, and the population

	Total		Subsample		Population
	Sample schools	Participating schools	Sample schools	Participating schools	
	% of *n* (601)	% of *n* (131)	% of *n* (261)	% of *n* (62)	% of *n* (6,309)
**School organization**					
Public school	76	77	72	73	77
Independent school	24	23	28	27	23
**Type of municipality**					
Metropolitan	16	13	17	15	14
Suburbs surrounding metropolitan areas	14	12	13	8	15
Larger cities and surrounding suburbs	36	40	35	40	34
Densely populated	10	9	11	15	10
Other municipalities	25	27	24	23	28
**School size**					
<60 students	38	44	39	45	69
60–99 students	30	30	31	29	19
≥100 students	32	26	30	26	12
**School grade**					
5	33	32	33	31	54
8	34	33	35	37	26
11	33	35	32	32	20

**Table 4:** Student-level characteristics in total sample, subsample, participating schools, and the population

	Total		Subsample		Population
	Sample students	Participating students	Sample students	Participating students	
	% of *n* (56,474)	% of *n* (3,477)	% of *n* (23,848)	% of *n* (1,305)	% of *n* (326,040)
***Individual information***					
**Parental education**					
Primary school/unknown	10	10	9	10	12
High school	51	50	50	48	51
University degree	40	40	42	43	37
**Household income**					
<450,000 SEK/unknown	32	32	30	33	35
450,000–649,999 SEK	29	31	29	31	30
≥650,000 SEK	40	37	41	36	35
**Birth country^a^**					
Sweden	76	79	77	78	74
Other country	24	21	23	23	26
**Sex**					
Female	48	53	49	56	48
Male	52	47	51	44	52
**School grade**					
5	17	35	18	33	34
8	34	35	37	36	33
11	50	31	46	30	33
***School-level information applied to students***					
**School organization**					
Public school	75	84	74	78	81
Independent school	25	17	26	22	19
**Type of municipality**					
Metropolitan	18	14	19	14	17
Suburbs surrounding metropolitan areas	18	14	18	9	17
Larger cities and surrounding suburbs	36	41	36	41	36
Densely populated	8	9	9	14	9
Other municipalities	20	23	18	21	22
**School size**					
<60 students	15	40	16	42	37
60–99 students	25	32	27	27	29
≥100 students	60	28	57	31	34

a Swedish: child and at least one parent born in Sweden. Other country: child or both parents born abroad.

The distribution of participating schools according to type of municipality was representative for the population at school level (Table 3). Students in metropolitan areas were however less likely to participate, while students in larger cities and surrounding suburbs were overrepresented both in the total and in the subsample. Furthermore, students in densely populated municipalities were somewhat overrepresented in the subsample (Table 4). Participating schools and students were distributed similarly between public and independent schools as compared to the population (Tables 3 and 4).

The distribution of parental income, education, and birth country were similar, although not identical, in participants and in the population (Table 4). The population consisted of slightly more males than females, while females to a higher degree participated in the survey. This was most pronounced in the subsample where 56% were females compared to 48% in the population.

## Discussion

The participation rate in this national dietary survey was high, 68%, in the total sample. In the subsample, participation was lower, 55%, probably because some students were deterred by blood and urine samples and the fact that written consent was needed from the legal guardians. Among those without biological sampling, 79% participated which was comparable to participation among children in school grade 5 in the latest national dietary survey Riksmaten Children 2003 (1). Conversely, in Riksmaten Adults 2010–2011, where a random population sample of 5,000 adults were invited, only 46% participated (2). The school-based design in Riksmaten Adolescents 2016–2017 and Riksmaten Children 2003 may be a contributing factor to the high participation rates. Recruiting on school level was more difficult with only about 20% of schools accepting the invitation. However, the desired number of classes was reached before all schools in the random sample could be contacted by phone and thus all non-participating schools did not have a chance to actively decline.

Even though slight differences were observed compared to the population, participants can be considered to be representative with regard to school organization; school size; and parental income, education, and background. Schools were distributed throughout Sweden and all 10 types of municipalities were represented. Overall, no major skewness was observed in the distribution of students according to type of municipality.

The survey employed standardized methods while introducing a new validated web-based method for dietary assessment and questionnaires. This method was well received among participants and the flexible interface provides a useful tool for future surveys. The substantial amount of information collected in the current survey will contribute to a wide variety of areas of food safety, policy-making, and healthy food habits. Results will also be presented as authority reports posted on the NFA website or as scientific articles in peer-reviewed journals.
